# The development of the ADO-SQ model to predict 1-year mortality in patients with COPD

**DOI:** 10.1177/02692163221080662

**Published:** 2022-03-24

**Authors:** Catherine Owusuaa, Cor van der Leest, Gea Helfrich, Roxane Heller-Baan, CJ van Loenhout, Jacobine W Herbrink, Daan Nieboer, Carin CD van der Rijt, Agnes van der Heide

**Affiliations:** 1 Department of Medical Oncology, Erasmus MC Cancer Institute, Rotterdam, The Netherlands; 2 Department of Pulmonary Diseases, Amphia Hospital, Breda, The Netherlands; 3 Department of Pulmonary Diseases, Maasstad Hospital, Rotterdam, The Netherlands; 4 Department of Pulmonary Diseases, Ikazia Hospital, Rotterdam, The Netherlands; 5 Department of Pulmonary Diseases, Admiraal De Ruyter Hospital, Goes, The Netherlands; 6 Department of Pulmonary Diseases, Van Weel Bethesda Hospital, Dirksland, The Netherlands; 7 Department of Public Health, Erasmus MC, Erasmus University Medical Center, Rotterdam, The Netherlands

**Keywords:** Chronic obstructive pulmonary disease, mortality determinants, nomogram, prediction model, surprise question

## Abstract

**Background:**

Goals of end-of-life care must be adapted to the needs of patients with chronic obstructive pulmonary disease (COPD) who are in the last phase of life. However, identification of those patients is limited by moderate performances of existing prognostic models and by limited validation of the often-recommended surprise question.

**Aim:**

To develop a clinical prediction model to predict 1-year mortality in patients with COPD.

**Design:**

Prospective study using logistic regression to develop a model in two steps: (1) external validation of the ADO, BODEX, or CODEX models (A = age; B = body mass index; C = comorbidity; D = dyspnea; EX = exacerbations; O = airflow obstruction); (2) updating of best performing model and extending it with the surprise question. Discriminative performance of the new model was assessed using internal-external validation and measured with area under the curve (AUC). A nomogram and web application were developed.

**Settings/participants:**

Patients with COPD from five hospitals (September–November 2017).

**Results:**

Of the 358 included patients (median age 69.5 years, 50% male), 63 (17%) died within a year. The ADO index (AUC 0.73) had the best discriminative ability compared to the BODEX (AUC 0.71) or CODEX (AUC 0.68), and was extended with the surprise question. The resulting ADO-surprise question (SQ) model had an AUC of 0.79.

**Conclusion:**

The ADO-SQ model offers improved discriminative performance for predicting 1-year mortality compared to the surprise question, ADO, BODEX, or CODEX. A user-friendly nomogram and web application (https://dnieboer.shinyapps.io/copd) were developed. Further external validation of the ADO-SQ in patient groups is needed.

**What is already known about the topic?**

- Advance care planning has benefits for patients with chronic obstructive pulmonary disease (COPD) who are in the last year of life.
- The identification of patients who have a high 1-year mortality probability is limited by the moderate performances of existing prognostic models and the limited validation of the often recommended surprise *question (Would you be surprised if this patient died in the next year?)*.

**What this paper adds**

- In a cohort of 358 patients, the ADO-surprise question (SQ) model that consists of predictors from the ADO model (age, dyspnea, and airflow obstruction) and the surprise question, had better discrimination in predicting 1-year mortality than the ADO model or surprise question alone.
- A nomogram and web application can be applied to calculate the 1-year mortality for individual patients with COPD.

**Implications for practice, theory or policy**

- Physicians can apply the ADO-SQ nomogram to identify patients who may benefit from advance care planning based on their 1-year mortality probability.
- The ADO-SQ nomogram could be applied in educational programs about advance care planning.

## Introduction

Chronic obstructive pulmonary disease (COPD) is a life-limiting disease and a major cause of morbidity and mortality globally.^1^ In 2015, COPD accounted for about 3.1 million deaths, which was 5% of all deaths globally.^2^ One-year mortality has been reported as low as 5% for patients without acute exacerbation in 1 year and as high as 43% for patients following an acute exacerbation of severe COPD.^3,4^ End-of-life care for these patients, which is defined as care in the last year before death, must be focused on improving quality of life by addressing patients’ supportive and palliative care needs.^5^ Such care includes advance care planning discussions between physicians and patients, to support patients in defining and sharing their personal values and preferences regarding medical care, especially when death approaches.^6,7^ Patients with COPD have more unmet palliative care needs compared to patients with (lung) cancer. Additionally, patients with COPD have been shown to receive insufficient care from end-of-life care services in the last year of life.^8,9^ There is some evidence that the relatively unpredictable illness trajectory of COPD is a barrier to adequate end-of-life care provision.^10^ Accurate prediction of mortality in patients with COPD could help physicians decide when to initiate advance care discussions, which could facilitate the initiating of palliative care.

Guidelines for end-of-life or palliative care worldwide often recommend the surprise question, and not disease-specific prognostic models, to identify patients who are approaching the end of life.^11,12^ The surprise question—Would you be surprised if this patient died in the next year?—involves the physician’s clinical prediction of survival.^13^ For patients with COPD, however, the performance of the surprise question has not been studied as extensively as for patients with cancer or renal failure. Indeed, a review by White et al.^14^ on the validation of the surprise question found only one study in patients with COPD. No information about the discriminative performance was reported.^15^

Several prognostic indexes and models consisting of various combinations of clinical predictors have been developed with the goal of predicting mortality in patients with COPD. A review by Bellou et al.^16^ reported 209 different prognostic models, in which the most commonly used predictors were age, airflow obstruction, smoking, body mass index, dyspnea, previous COPD exacerbations, previous hospital admission, or comorbidity. Despite the abundance of prognostic models, most models have not been sufficiently validated internally or externally.^16^ The updated ADO (age, dyspnea, and airflow obstruction) and BODE (body mass index, airflow obstruction, dyspnea, and exercise capacity) models have been studied most extensively.^17^ However, these models were mostly shown to have a moderate discriminative ability performance (AUC or c-statistic ranging between 0.6 and 0.8).^16,18^

Although there are several prognostic models for COPD, they have not been studied together with physician’s clinical prediction. It is well known that prediction is most accurate when prognostic factors are combined with physician’s clinical prediction.^19,20^ We aimed to assess whether an integrated clinical model, which combines known predictors with the surprise question, improves 1-year mortality prediction.

## Methods

### Study design and patients

We prospectively included patients from the Pulmonary Diseases departments and outpatient clinics in five hospitals in the Netherlands: two general (Van Weel Bethesda Hospital and Admiraal De Ruyter Hospital) and three teaching hospitals (Amphia Hospital, Maasstad Hospital, and Ikazia Hospital). Patients (⩾18 years) with a diagnosis of COPD according to Global Initiative for Chronic Obstructive Lung Disease (GOLD) classification were eligible for inclusion.^21^ Patients with a combination of COPD and asthma were excluded. Patients were assessed for participation using the in- and exclusion criteria by the participating pulmonologists. The Medical Research Committee of the Erasmus MC, University Medical Center Rotterdam, and the institutional review boards of the participating hospitals approved the study (MEC-2017-289). All participating patients were informed about the study before they gave their informed consent.

Potential predictors to be collected were the surprise question (“Would you be surprised if this patient died in the next year”) and predictors from the validated updated ADO, BODEX, and CODEX models (Box 1)^16^: dyspnea was assessed on the Medical Research Council scale (MRC; ranging from 0 = no breathlessness to 5 = too breathless to leave the house)^22^ reported by the patient; airflow obstruction was measured using the forced expiratory volume in 1 second (FEV_1_); exacerbations were defined as the requirement of hospitalizations in the previous year; and comorbidity was assessed with the Charlson comorbidity index (defined as the occurrence of any comorbidity except chronic pulmonary disease).^23^ The study size was estimated at 300 patients, which was based on an average mortality rate of 20% and the number of expected deaths (60) in relation to the number of predictors (4–5).^3,4,24^ For each included patient, the attending medical specialist or resident answered the surprise question and rated the dyspnea level. Information on other predictors was obtained from patients’ medical records.

**Box 1.** Prognostic models for mortality in patients with COPD.

Prognostic model	Predictors	No. of datasets
ADO	Age, Dyspnea, Airflow obstruction	11
BODE	Body mass index, Airflow obstruction, Dyspnea, Exercise capacity	8
BODEX	Body mass index, Airflow obstruction, Dyspnea, Exacerbations	4
CODEX	Comorbidity, Airflow obstruction, Dyspnea, Exacerbations	3
DOSE	Dyspnea, Airflow obstruction, Smoking, Exacerbations	3

We followed all patients for a maximum of 1 year and collected information on their vital status from medical records or by telephone contact with their general practitioner. For patients who had died or were alive at 1 year, respectively, the date of death or the date of the last contact with the attending physician was noted.

### Statistical analysis

The primary outcome was all-cause 1-year mortality. We assessed sensitivity, specificity, positive predictive value, and negative predictive value of the surprise question. We externally validated the ADO, BODEX, and CODEX model in our cohort. We assessed discriminative ability of these models using the are under the receiver operating characteristic (AUC), whereby 0.5 means no discriminative ability of the model and 1 means perfect discrimination.^25^ Calibration of the models were assessed graphically. Subsequently we updated the best performing model in our cohort by re-estimating the regression coefficients, while including the surprise question as additional predictor. Missing values were considered to be missing at random and were imputed using multiple imputation. The results on each of the analyses on the imputed datasets were pooled using Rubin rules.^26^ Sensitivity analyses were performed to investigate the potential impact of the violation of missing at random assumption by assuming that patients who had missing FEV_1_ would score on average lower. To investigate heterogeneity in model performance across the different hospitals, we performed an internal-external validation of the developed model.^27^ During this analysis, the updated model was fitted with data from all participating hospitals except one, and then validated with data from the excluded hospital. This was repeated until the model was validated for each hospital once. The model’s discriminative performance at 1 year was evaluated using the AUC.

We developed a nomogram and a web application to enable easy calculation of the probability of death at 1 year based on our model. All statistical analyses were performed with R statistical software (version 3.6.0.), using the *mice* package for imputation and the *R Shiny* package for the web application. A *p*-value <0.05 was considered to be significant. The manuscript was structured using the guidelines of the Transparent reporting of a multivariable prediction model for individual prognosis or diagnosis (TRIPOD) checklist.^28^

## Results

**Figure 1.**
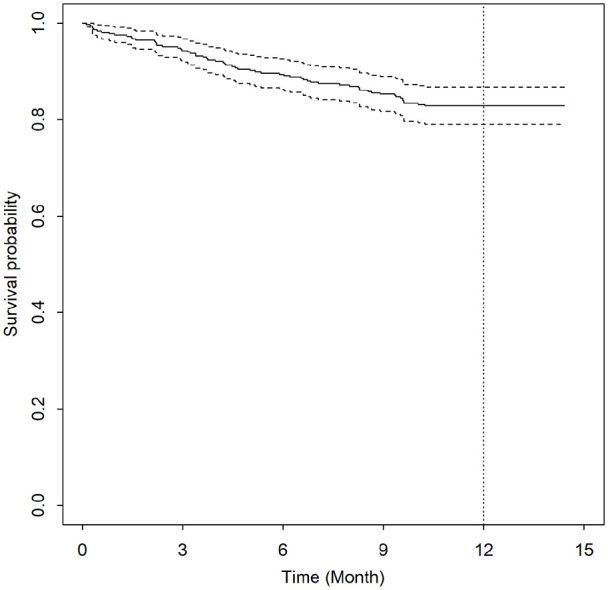
Kaplan-Meier survival curve among 358 patients with chronic obstructive pulmonary disease.

Three hundred and fifty-eight patients with COPD were included between September 28, 2017, and November 24, 2017. Their median age was 69.5 years, and 50% of the patients were male. Patients’ characteristics are listed in Table 1, Supplemental Table E1, and E2). Most patients presented with COPD GOLD II (40.5%) or GOLD III (31.6%). At 1-year follow-up, 62 (17.3%) patients had died. The trend in survival is shown in Figure 1.

**Table 1.** Patient characteristics.

	Overall (*N* = 358)	Missing (%)
Patients per hospital		0.0
Ikazia Hospital	97 (27.1)	
Maasstad Hospital	160 (44.7)	
Amphia Hospital	30 (8.4)	
Van Weel Bethesda Hospital	47 (13.1)	
Admiraal De Ruyter Hospital	24 (6.7)	
Setting		0.0
Inpatient	226 (63.1)	
Outpatient	132 (36.9)	
Age, years (median [IQR])	69.5 [63.0–76.0]	0.0
Sex (%)		0.0
Female	179 (50.0)	
Body mass index, kg/m^2^ (median [IQR])	25 [21–29]	3.2
Comorbidity, Charlson score (median [IQR])	2 [1–11]	0.0
GOLD classification (%)		0.0
I	19 (5.3)	
II	145 (40.5)	
III	113 (31.6)	
IV	78 (21.8)	
Unknown	3 (0.8)	
FEV_1_, % predicted (median [IQR])	49 [36–62]	6.3
Dyspnea, MRC grade (%)		0.0
0	11 (3.1)	
1	61 (17.0)	
2	70 (19.6)	
3	69 (19.3)	
4	73 (20.4)	
5	74 (20.7)	
No. of acute exacerbations* (%)		0.0
0	279 (77.9)	
1	47 (13.1)	
2	16 (4.5)	
3	12 (3.4)	
4	3 (0.8)	
5	1 (0.3)	
Surprise question (%)		2.2
“No” (reference “Yes”)	109 (30.4)	
Death at 1-year follow-up	62 (17.3)	0.0

FEV_1_: forced expiratory volume in 1 second; GOLD: Global Initiative for Chronic Obstructive Lung Disease; IQR: interquartile range; MRC: Medical Research Council; No.: Number.

* Number of acute exacerbations in the previous year. An acute exacerbation during the inclusion time was not included for analysis.

The surprise question was mostly answered by the attending medical specialist (79%). A negative answer to the surprise question had a sensitivity of 63% (95% CI 50–75), specificity of 76% (95% CI 71–81), positive predictive value of 36% (95% CI 30–42), and negative predictive value of 91% (95% CI 88–93) for 1-year mortality. The prognostic accuracy was 74% and did not significantly differ whether it was answered by the attending medical specialist or a resident (*p* = 0.6456). The AUC for prediction of 1-year mortality using the surprise question was 0.70 (95 CI 0.63–0.76).

The ADO model had the best AUC of 0.73, compared to the BODEX (AUC 0.71) and the CODEX index (AUC 0.68), and thus the best-performing model to extend with the surprise question. This resulted in the ADO-surprise question (SQ) model, which consisted of the following predictors: age, dyspnea level, FEV_1_, and surprise question. The univariable and multivariable logistic regression analyses of those predictors are presented in Table 2. Although approximately two-third of our cohort were inpatients, a previously hospitalization with an COPD exacerbation was not significant for mortality (HR 1.74; CI 0.94, 3.20). For the internal-external validation or calibration of the ADO-SQ, we merged data of the two hospitals with the lowest inclusion number (Amphia Hospital and Admiraal De Ruyter Hospital). The ADO-SQ model had improved discrimination with an AUC of 0.79 (95% CI 0.73–0.85) (Table 3). The ADO-SQ was reasonably calibrated per participating hospital (AUC ranging from 0.78 to 0.83), except for the two merged hospitals (AUC 0.55), for which the model showed poorer calibration (Supplemental Table E3 and Figure E1 in the online data supplement). The performances of the surprise question and the ADO-SQ were similar across in- and outpatients (Supplemental Table E4). A sensitivity analysis is presented in Supplemental Table E5.

**Table 2.** Univariable and multivariable analyses of predictors in the ADO-SQ model.

	Univariable analysis HR (95% CI)	*p*-value	Multivariable analysis HR (95% CI)	*p*-value
Surprise question (“No”)	5.47 (3.06–9.79)	<0.001	2.93 (1.51–5.69)	0.016
Age (per decade)	2.10 (1.51–2.91)	<0.001	1.72 (1.20–2.47)	0.003
FEV_1_ (per 10% decrease)	1.75 (1.40–2.19)	0.106	1.04 (0.87–1.25)	0.688
Dyspnea (per point increase on MRC)	1.14 (0.97–1.33)	<0.001	1.37 (1.05–1.78)	0.020

CI: confidence interval; FEV_1_: forced expiratory volume in 1 second; HR: hazard ratio; MRC: Medical Research Council.

**Table 3.** Discriminative performance for the surprise question, ADO, BODEX, CODEX, and ADO-SQ.

Prognostic model	1-year AUC (95% CI)
Surprise question	0.70 (0.63–0.76)
Updated ADO	0.73 (0.67–0.80)
BODEX	0.71 (0.65–0.77)
CODEX	0.68 (0.61–0.75)
ADO-SQ	0.79 (0.73–0.85)

**Figure 2.**
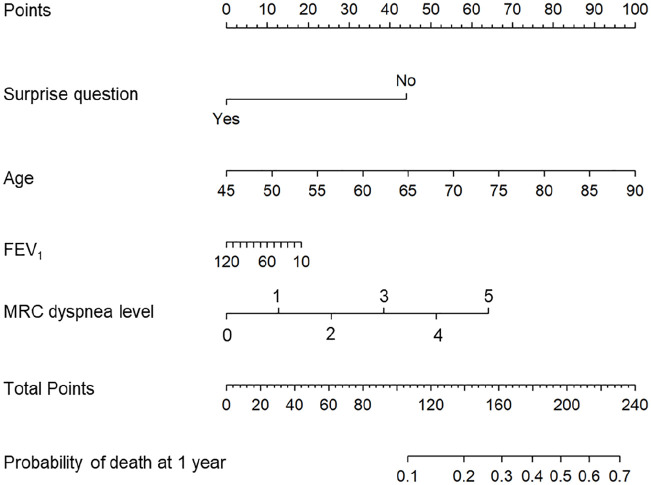
Nomogram to predict 1-year mortality with the ADO-SQ model. FEV_1_: forced expiratory volume in 1 second; MRC: Medical Research Council; Surprise question: “Would you be surprised if this patient died in the next year?” Instructions for use: locate the answer to the surprise question; draw a straight upwards to the Points axis. Repeat this for the other predictors: age, FEV_1_, and MRC dyspnea level. Sum the points for all predictors on the Total Points axis. Draw a line straight down to the Probability of death at 1-year axis to find the patient’s probability of dying within 1 year.

A nomogram (Figure 2) and a web application (https://dnieboer.shinyapps.io/copd) are available to calculate the probability of death at 1 year for COPD patients. See Box 2 for the formulas of the SQ and ADO-SQ models and a patient example with the calculated 1-year mortality probability.

**Box 2.** Model formulas and patient example.

**SQ model:** Lp = −2.31 + 1.74 × {SQ = NO} P(1 year mortality) = 1/(1 + exp(-lp)) **ADO model:** Lp = −6.71 + 1.16 × {SQ = NO} + 0.05 *Age + 0.29 * dyspnea – 0.03 * fev/10 P(1 year mortality) = 1/(1 + exp(-lp)) **Patient example:** Male patient Surprise question: No Age: 74 years Dyspnea: MRC scale 5 FEV_1_ (% of predicted): 25% *1-year mortality probability according to*: SQ: 36% ADO-SQ: 45%

## Discussion

### Main findings

This is to our knowledge the first study to integrate an existing prognostic model with the surprise question to evaluate the likelihood of dying within 1 year for patients with COPD. The resulting ADO-SQ model, which is based on predictors of the ADO model (age, dyspnea level, and FEV_1_) and the surprise question, improved the prediction of 1-year mortality in our sample compared to the ADO or the surprise question alone. The ADO-SQ model has several qualities, which make its application easier compared to other models. First, the ADO-SQ is an extension of the well-validated ADO model, which was not only the best-validated model in our cohort, but also in other cohorts.^16,29^ Second, the ADO-SQ consists of four predictors that are often readily obtainable in clinical practice. Thus, the ADO-SQ can be applied in both primary and secondary care, that is, at the general practices and hospitals respectively. Although several other prognostic models for COPD also consist of just a few predictors, some predictors may need extra efforts, such as calculation of the Charlson comorbidity index score for the CODEX model and the performance of the 6-minute walking test for the BODE model. Lastly, unlike most prognostic models that were examined in outpatient cohorts, the ADO-SQ was developed in both in- and outpatient cohorts, which makes its use in both settings applicable.^16^

Although our model is unique in the sense that it integrates a validated prognostic model for COPD with the surprise question, it is not the first model to include the surprise question. The ProPal-COPD model by Duenk et al.^30^ included the surprise question with six other variables to predict 1-year mortality in 155 patients who had been hospitalized for acute exacerbation. Those predictors were the dyspnea level, FEV_1_, body mass index, previous hospitalizations for acute exacerbation, Clinical COPD questionnaire, and specific comorbidities. The discriminative ability of this model was 0.82 AUC. Some differences between the ProPal-COPD and the ADO-SQ may make the latter model more attractive. First, the number of variables of the ProPal-COPD (11 predictors) may make it more complex to use than the four variables of the ADO-SQ. Second, The ProPal-COPD may also have been over fitted due to the number of deaths in relation to the number of predictors (30/11) compared to the ADO-SQ (62/4).^24,31^ Third, unlike the predictors of the ADO-SQ, those of the ProPal-COPD were dichotomized, which is not ideal for the discriminative performance due to possible loss of information. Lastly, although both ProPal-COPD and ADO-SQ await external validation, the internal-external validation analysis applied for the ADO-SQ demonstrates some level of its external validity.^27^

Of note, results of our study showed a poor to modest predictive performance of the surprise question. Research evaluating the surprise question has been very limited in patients with COPD. However, the poor positive predictive value (PPV) of 36.0% of a negative answer to the surprise question in our cohort was similar to that found in cohorts of patients with renal failure (38.5%).^14^ Moreover, the surprise question has only shown a relatively high PPV in patients with cancer (49.5%).^14^ The surprise question might thus be more difficult to answer for patients with COPD than for patients with cancer. The poor performance and subjectivity of the surprise question may cause variability in the discriminative performance of the ADO-SQ model in other cohorts and settings. Studying the surprise question together with the predictors of the ADO, that is, on the same questionnaire, may improve on the performance of the surprise question alone.

### Implications for policy, clinical practice, and research

The application of the ADO-SQ model by physicians is facilitated with the development of a nomogram and web-application. A patient’s probability of death at 1 year could aid physicians in timely focusing on the initiation of advance care planning discussions, which could facilitate the initiation of palliative care and advance care planning. Furthermore, the ADO-SQ nomogram could be applied in educational programs about advance care planning.

### Future considerations

Future studies should externally validate the ADO-SQ model, in another group of COPD patients and evaluate its association and feasibility with palliative care. Additionally, further research could explore the performance of other prognostic models (Box 1) or predictors integrated with the surprise question.

### Strengths and limitations

An important strength of our study is the development of the nomogram and web application, which will facilitate use in clinical practice. Additionally, the fact that patients were recruited in five different hospitals adds some level of heterogeneity and generalizability to our study. However, our study has some limitations. First, it does not provide information on the interpretation of the mortality probability, for example, at which probability the model is most sensitive, which could be difficult for physicians. Second, due to the relatively low mortality rate (17.3%), possibly due to a relatively high percentage of patients with COPD GOLD I or II (45.8), our model might excel in that group or underperform in more severely ill patients (GOLD III or IV). The relatively low mortality rate limited the possibility to analyze a subgroup of severely ill patients. The distribution of patients may also explain why the severity of FEV_1_ impairment was not significant for 1-year mortality. Although the number of missing FEV1 was relatively low, we found no differences between patients with and patients without missing FEV1, and a sensitivity analyses showed that FEV remained not significant (Supplemental Tables E2 and E5). Third, we did not report the cause of death, whether it was due to respiratory or non-respiratory causes. Fourth, apart from the ADO, BODEX, and CODEX, we did not externally validate other well-validated prognostic models such as the BODE or DOSE (Box 1). This was partly due to insufficient data on exercise capacity (6-minute walking test) or smoking in our cohort. Fifth, the ADO-SQ was developed in mainly inpatients, which may limit the generalizability to outpatients in another study population. However, we found no major differences between our in- and outpatient populations and the performance of the ADO-SQ was similar in both populations (Supplemental Tables E1 and E4). Therefore, our sample with in- and outpatients with different severity of COPD (i.e. GOLD I-IV) may be representative of a wider and international patient group. An external validation of the developed model is warranted to assess its generalizability. Lastly, the internal-external validation of the ADO-SQ showed that its performance was relatively stable across the larger hospitals, but poorer in the two smallest hospitals.

## Conclusion

We conclude that the ADO-SQ model, which is an integration of the well-validated ADO model with the surprise question, has improved discriminative performance in predicting 1-year mortality in patients with COPD compared to the surprise question, ADO, BODEX, or CODEX models. The available nomogram and web application can be useful to physicians in decision-making about the initiation of advance care planning discussions, which could facilitate palliative care. However, before widespread application of the ADO-SQ model can be recommended, it needs to be validated in external cohorts.

## Supplemental Material

sj-pdf-1-pmj-10.1177_02692163221080662 – Supplemental material for The development of the ADO-SQ model to predict 1-year mortality in patients with COPD Click here for additional data file. Supplemental material, sj-pdf-1-pmj-10.1177_02692163221080662 for The development of the ADO-SQ model to predict 1-year mortality in patients with COPD by Catherine Owusuaa, Cor van der Leest, Gea Helfrich, Roxane Heller-Baan, CJ van Loenhout, Jacobine W Herbrink, Daan Nieboer, Carin CD van der Rijt and Agnes van der Heide in Palliative Medicine
