# Supplementary material for: The development of the ADO-SQ model to predict 1-year mortality in patients with COPD
Source: Palliat Med. 2022 Mar 24;36(5):821–9. doi: 10.1177/02692163221080662 (PMC9087317; doi:10.1177/02692163221080662)
Supplement: sj-pdf-1-pmj-10.1177_02692163221080662 – Supplemental material for The development of the ADO-SQ model to predict 1-year mortality in patients with COPD [file sj-pdf-1-pmj-10.1177_02692163221080662.pdf]

Online Data Supplement

The development of the ADO-SQ model to predict 1-year mortality in patients with COPD

Catherine Owusuaa, MD; Cor van der Leest, MD, PhD; Gea Helfrich, MD; Roxane Heller-Baan, MD; C.J. van Loenhout, MD; Jacobine W. Herbrink, MD; Daan Nieboer, MSc; Carin C.D. van der Rijt, MD, PhD; Agnes van der Heide, MD, PhD

Table E1. Patient characteristics for the inpatient and outpatient populations

	Inpatients *N*= 84	Outpatients *N*= 274	p-value
Age, yr (median [IQR])	69.5 [64.0-80.0]	69.5 [63.0-75.0]	0.195
Sex, female (%)	48 (57.1)	131 (47.8)	0.170
Body mass index, kg/m2 (median [IQR])	25 [21-30]	25 [22-29]	0.707
Presence of ≥1 comorbidity (%)	13 (15.5)	39 (14.2)	0.916
FEV_1, % predicted (median [IQR])	43 [32-57]	50 [37-64]	0.010
Dyspnea, MRC grade (%)			<0.001
0	0 (0.0)	11 (4.0)	
1	7 (8.3)	54 (19.7)	
2	8 (9.5)	62 (22.6)	
3	14 (16.7)	55 (20.1)	
4	22 (26.2)	51 (18.6)	
5	33 (39.3)	41 (15.0)	
No. of acute exacerbations* (%)			<0.001
0	53 (63.1)	226 (82.5)	
1	12 (14.3)	35 (12.8)	
2	8 (9.5)	8 (2.9)	
3	8 (9.5)	4 (1.5)	
4	2 (2.4)	1 (0.4)	
5	1 (1.2)	0 (0.0)	
Surprise question (%)			
‘No’ (reference ‘Yes’)	37 (44.0)	72 (26.3)	0.003
Death at 1-year follow-up	26 (31.0)	36 (13.1)	<0.001

FEV1: forced expiratory volume in 1 second; GOLD: Global Initiative for Chronic Obstructive Lung Disease; IQR: interquartile range; MRC: Medical Research Council; No.: Number

*: Number of acute exacerbations in the previous year. An acute exacerbation during the inclusion time was not included for analysis.

Table E2. Patient characteristics of patients with missing FEV_1 and no missing FEV_1

	Missing FEV_1 *N*= 22	No missing FEV_1 *N*= 336
Age, yr (median [IQR])	67.5 [61.5-72.8]	70.0 [64.0-76.0]
Sex, female (%)	8 (36.4)	171 (50.9)
Body mass index, kg/m2 (median [IQR])	27 [23-28]	25 [22-29]
Presence of ≥1 comorbidity (%)	4 (18.2)	48 (14.3)
FEV_1, % predicted (median [IQR])	NA [NA, NA]	49 [36-62]
Dyspnea, MRC grade (%)		
0	1 (4.5)	10 (3.0)
1	6 (27.3)	55 (16.4)
2	3 (13.6)	67 (19.9)
3	4 (18.2)	65 (19.3)
4	4 (18.2)	69 (20.5)
5	4 (18.2)	70 (20.8)
No. of acute exacerbations* (%)		
0	17 (77.3)	262 (78.0)
1	3 (13.6)	44 (13.1)
2	2 (9.1)	14 (4.2)
3	0 (0.0)	12 (3.6)
4	0 (0.0)	3 (0.9)
5	0 (0.0)	1 (0.3)
Surprise question (%)		
'No' (reference 'Yes')	8 (36.4)	101 (30.1)
Death at 1-year follow-up	4 (18.2)	58 (17.3)

Table E3. Internal-external validation of the ADO-SQ per hospital

Hospital	AUC (95% CI)
Ikazia Hospital	0.83 (0.73, 0.93)
Maasstad Hospital	0.78 (0.71, 0.86)
Amphia Hospital + Admiraal De Ruyter Hospital	0.55 (0.31, 0.78)
Van Weel Bethesda Hospital	0.82 (0.69, 0.96)

ADO-SQ: Age, Dyspnea, FEV_1, surprise question; AUC: area under the curve; FEV_1: forced expiratory volume in 1 second

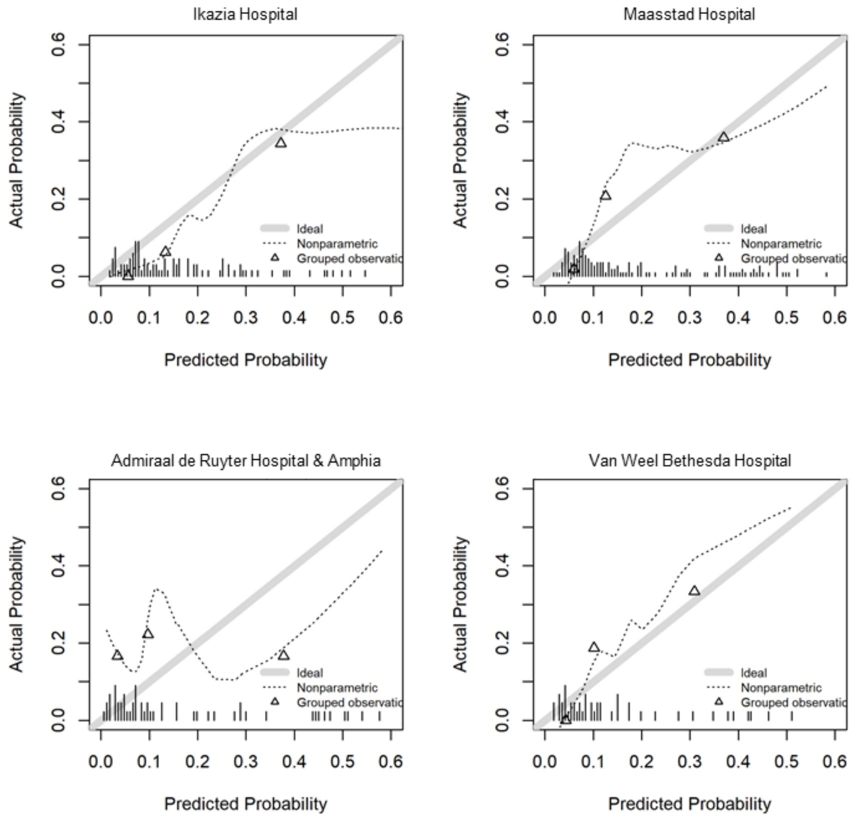

Figure E1. Calibration of the ADO-SQ model per hospital

Table E4. Discriminative performance for the inpatient and outpatient populations

Model	Inpatients AUC (95% CI)	Outpatients AUC (95% CI)	p-value
Surprise question	0.68 (0.57-0.79)	0.68 (0.60-0.77)	0.867
ADO-SQ	0.77 (0.68-0.87)	0.75 (0.66-0.84)	0.904

ADO-SQ: Age, Dyspnea, FEV_1, surprise question; AUC: area under the curve

Table E5. Sensitivity analysis

Predictor	Coefficient	S.E.	p-value
Intercept	-6.7726	1.3152	<0.0001
Surprise question	1.0728	0.3405	0.0016
Age	0.5383	0.1840	0.0034
FEV_1	0.0329	0.0884	0.7096
Dyspnea	0.3172	0.1332	0.0172

FEV_1: forced expiratory volume in 1 second
